# Transcriptomic analysis revealed increased expression of genes involved in keratinization in the tears of COVID-19 patients

**DOI:** 10.1038/s41598-021-99344-3

**Published:** 2021-10-06

**Authors:** Leonardo Mastropasqua, Lisa Toto, Luigi Chiricosta, Francesca Diomede, Agnese Gugliandolo, Serena Silvestro, Guya Diletta Marconi, Bruna Sinjari, Jacopo Vecchiet, Francesco Cipollone, Damiano D’Ardes, Antonio Auricchio, Manuela Lanzini, Sergio Caputi, Rossella D’Aloisio, Emanuela Mazzon, Oriana Trubiani

**Affiliations:** 1 Ophthalmic Clinic, Department of Medicine and Science of Ageing, University “G. d’Annunzio” of Chieti-Pescara, Via dei Vestini, 31, 66100 Chieti, Italy; 2 IRCCS Centro Neurolesi “Bonino-Pulejo”, 98124 Messina, Italy; 3 Department of Medical, Oral and Biotechnological Sciences, University “G. d’Annunzio” of Chieti-Pescara, 66100 Chieti, Italy; 4 Department of Innovative Technologies in Medicine and Dentistry, University “G. d’Annunzio” of Chieti-Pescara, 66100 Chieti, Italy; 5 Clinic of Infectious Diseases, Department of Medicine and Science of Aging, University “G. d’Annunzio” of Chieti-Pescara, 66100 Chieti, Italy; 6 Department of Medicine and Science of Aging, Clinica Medica Institute, University “G. d’Annunzio” of Chieti-Pescara, 66100 Chieti, Italy

**Keywords:** Diagnostic markers, Predictive markers, Prognostic markers, Viral infection, Gene expression, Gene regulation

## Abstract

Recent studies have focused their attention on conjunctivitis as one of the symptoms of coronavirus disease 2019 (COVID-19). Therefore, tear samples were taken from COVID-19 patients and the presence of SARS-CoV-2 was evidenced using Real Time reverse transcription polymerase chain reaction. The main aim of this study was to analyze mRNA expression in the tears of patients with COVID-19 compared with healthy subjects using Next Generation Sequencing (NGS). The functional evaluation of the transcriptome highlighted 25 genes that differ statistically between healthy individuals and patients affected by COVID-19. In particular, the NGS analysis identified the presence of several genes involved in B cell signaling and keratinization. In particular, the genes involved in B cell signaling were downregulated in the tears of COVID-19 patients, while those involved in keratinization were upregulated. The results indicated that SARS-CoV-2 may induce a process of ocular keratinization and a defective B cell response.

## Introduction

Coronavirus disease 2019 (COVID-19) is caused by SARS-CoV-2 infection. SARS-CoV-2 is a single-stranded RNA virus positive sense^1^ and was identified in December 2019 in Wuhan, and it is a novel RNA virus that is part of the family Coronaviridae^2,3^.

SARS-COV-2 has different proteins on envelopes: Spike, Envelope, Membrane and Nucleocapsid. Spike protein is responsible for the virus entry into the host cell. COVID-19 can affect individuals with mild symptoms: dry cough, fever, headache, dyspnea. Frequently more serious symptoms are found that can cause the onset of chronic pneumonia and septic shock^4^.

Ophthalmologists have performed an analysis that revealed the SARS-CoV-2 infection in a team of specialists that visited COVID-19 patients wearing N95 masks only as personal protective equipment (PPE). A few days before the onset of common symptoms such as pneumonia, they claim to have had inflammation of the conjunctiva^5^.

The receptor target of Spike protein is the angiotensin-converting enzyme 2 (ACE-2)^6^. The epithelial cells of both the cornea and conjunctiva showed the presence of ACE-2^7^.

One of the unusual symptoms that could be the first warning bell of SARS-COV-2 infection is conjunctivitis^8^.

Colavita et al. analyzed the case of a COVID-19 patient who returned to Italy from Wuhan who showed the first symptoms of dry cough, sore throat and bilateral conjunctivitis. It has been carried out an ocular swab demonstrating the presence of SARS-CoV-2 in the tear sample^9^.

These events could demonstrate the entry of the virus not only through the respiratory tract, but also through the mucous membranes of the eye^5^ and also SARS-CoV-2 could potentially be transmitted by conjunctival tears or secretions^10–14^.

A recent study found the presence of SARS-CoV-2 in the tears of COVID-19 patients with conjunctivitis, using the RT-PCR technique^15^.

The aim of the current study was to describe the transcriptional changes related to SARS-COV-2 viral infection, evaluating the up/down-regulation of mRNA in tears of hospitalized patients diagnosed with COVID-19 compared to healthy controls.

## Results

The demographic and clinical characteristics of the patients suffering from COVID-19 enrolled are summarized in Tables 1 and 2. The mean age of control group was 73.5 ± 10.7 years (75% males; 25% females). No statistical difference was found between COVID-19 and healthy groups in terms of age, gender and race (*p* > 0.05).

**Table 1:** Demographic and clinical characteristics of patients with COVID-19.

Variables	COVID-19 patients
Age (years)	74.5 ± 17.5
**Sex**	
Male	12 (65.0%)
Female	7 (35.0%)
**Race**	
Caucasian	19 (100%)
Comorbilities	16 (84.2%)
Cardiological	3 (15.8%)
Neurological	4 (21%)
Nephrological	5 (26.3%)
Autoimmune	0 (0%)
Hypertension	6 (31.6%)
Smoke tobacco	0 (0%)
Allergies	0 (0%)
Obesity	1 (5.7%)
Diabetes mellitus	3 (15.8%)
Others	11 (57.9%)

**Table 2:** Medical history and clinical characteristics of patients with COVID-19.

Variables	COVID-19 patients
**Symptoms**	
Dyspnea	17 (89.5%)
Dry cough	8 (42.1%)
Pneumonia	15 (78.9%)
Fever	11 (57.9%)
Myalgia	1 (5.3%)
Home therapy	15 (78.9%)
Antihypertensive/cardiovascular therapy	12 (63.2%)
Anticoagulants	3 (21.1%)
5-Alpha reductase inhibitors	1 (5.3%)
Anti-Psychotics/anxiolitics	4 (26.4%)
Oral Hypoglicemic drugs/insuline	2 (10.6%)
Hypouricemic drugs	1 (5.3%)
Duration of hospitalization (days)	26 ± 12
**Hospital therapy**	
Hydroxychloroquine	9 (47.4%)
Heparin	2 (10.5%)
Diuretics	1 (5.3%)
Anti-virals	10 (52.6%)
Steroids	2 (10.5%)
Antibiotics	4 (21.0%)
**Blood analysis at sampling day**	
Anemia	9 (47.4%)
Hyponatremia	2 (10.5%)
Hypernatremia	1 (5.3%)
PCR > 8 mg/l	10 (52.6%)
Lymphocytopenia	6 (31.6%)
Hyperkalemia	1 (5.3%)
Hypokalemia	2 (10.5%)
PCT > 0.15 ng/ml	2 (10.5%)
LDH > 222 U/l	1 (5.3%)
D-dimer > 0.5 mcg/ml	4 (21.0%)
Ferritin > 307 mcg/l	1 (5.3%)
Hypoalbuminemia	1 (5.3%)
Neutrophilia	1 (5.3%)
Creatinine > 1.3 mg/dl	3 (15.8%)
Mean Sa 02 at admission	91.9 ± 3.4%
Mean Sa 02 at sampling day	94.4 ± 3.1%
Mean Sa 02 at discharge	98.3 ± 0.6%
Bilateral conjunctivitis	7 (36.8%)
Before admission	3 (15.8%)
At admission	4 (21.0%)
At discharge	0 (0%)
Ventilation at sampling day	8 (42.1%)
Invasive	0 (0%)
Not invasive	8 (42.1%)
**Outcome**	
Recovery	7 (36.8%)
Death	12 (63.2%)

### RNA-seq analysis between healthy individuals against Covid-19 patients

**Figure 1:**
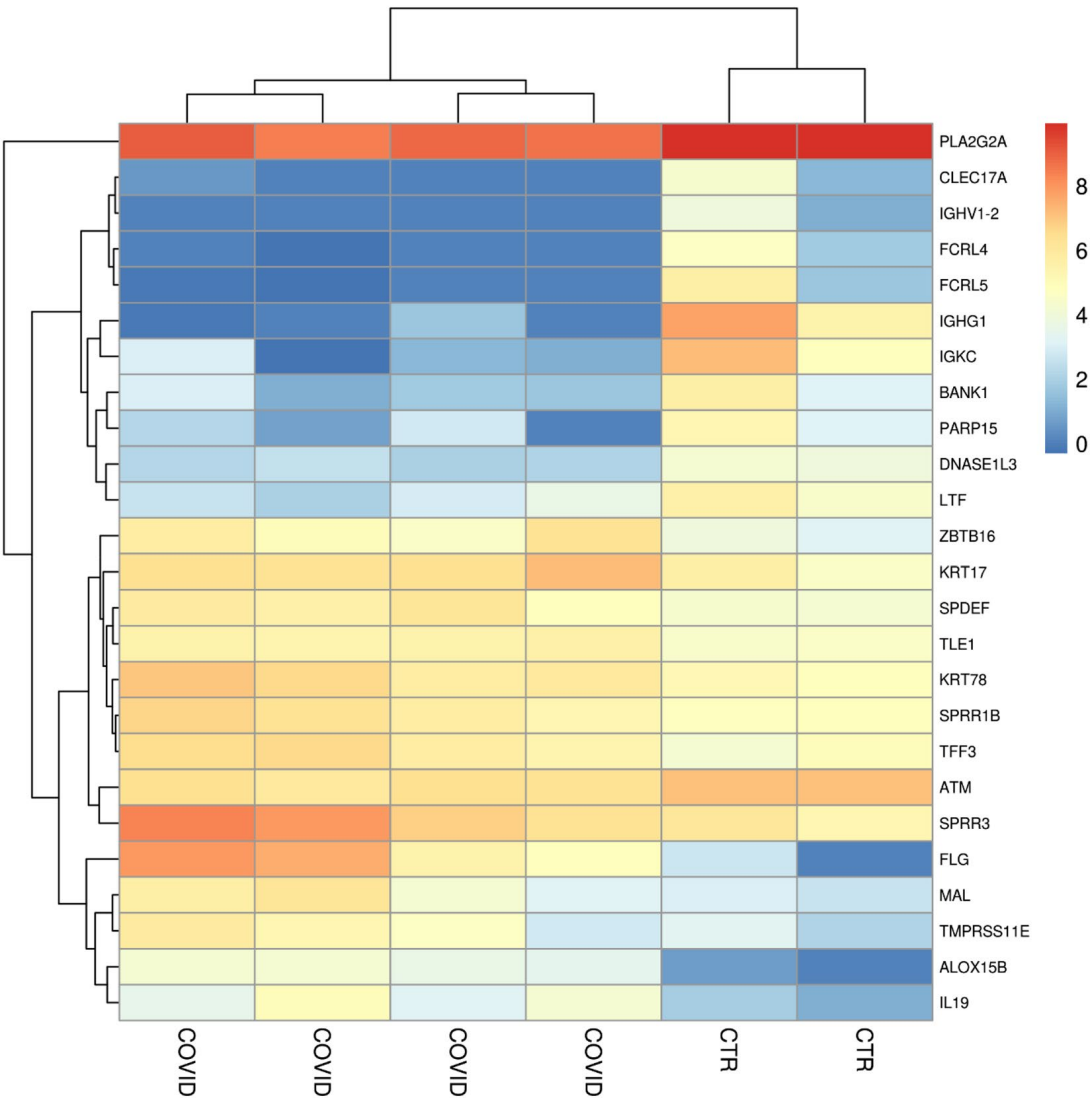
Heatmap of the differentially expressed genes between all the individuals. The heatmap represent the genes differentially expressed after the RNA-seq analysis was concluded. Moreover, the logarithmic correction made on the counts allows to appreciate the differences between the samples. As expected, the dendogram on the columns shows the closest similarity between the healthy subjects each other (CTR) and between the COVID-19 patients themselves (COVID).

The RNA-seq analysis revealed 25 genes that differ statistically between healthy individuals and COVID-19 patients (Table 3). Among them, 13 genes were upregulated while 12 genes were downregulated. Interestingly, 20 genes, the most of them, had more than a twofold change. Anyway, the remaining 5 genes had a onefold change. The changes in the behavior of the differential expressed genes between all the samples was depicted in the heatmap in Fig. 1. As expected, the dendogram obtained by the plot put the closest association between the control individuals themselves and the COVID-19 patients themselves. Furthermore, in order to inspect the role of the up- and down-regulated differentially expressed genes, the PANTHER Classification System^16^ for the Gene Ontology Biological Processes was used. The enriched Gene Ontologies of the Biological Processes obtained by PANTHER (Table 4) highlighted the “Keratinization” (False Discovery Rate (FDR) = 1.83 × 10^–3^) for the up- and the “Regulation of B cell activation” (FDR = 1.49 × 10^–3^), “Negative regulation of immune system process” (FDR = 2.21 × 10^–3^) and “Regulation of inflammatory response” (FDR = 1.64 × 10^–3^) for the down-regulated genes.

**Table 3:** Differentially expressed genes between healthy individual and Covid-19 patients.

Gene symbol	Gene name	Healthy expression	COVID-19 expression	Fold change	q-value
*ALOX15B*	Arachidonate 15-lipoxygenase type B	0.98	52.67	6.10	9.62e−04
*ATM*	ATM serine/threonine kinase	1270.92	548.16	− 1.21	3.45e−02
*BANK1*	B cell scaffold protein with ankyrin repeats 1	162.40	8.81	− 4.17	5.46e−03
*CLEC17A*	C-type lectin domain containing 17A	40.12	0.45	− 6.16	4.09e−02
*DNASE1L3*	Deoxyribonuclease 1 like 3	60.56	9.04	− 2.72	2.51e−02
*FCRL4*	Fc receptor like 4	57.02	0.18	− 7.37	2.60e−03
*FCRL5*	Fc receptor like 5	149.79	0.41	− 8.11	7.06e−05
*FLG*	Filaggrin	7.39	1308.95	7.49	4.99e−02
*IGHG1*	Immunoglobulin heavy constant gamma 1 (G1m marker)	1285.85	1.55	− 9.59	1.13e−03
*IGHV1-2*	Immunoglobulin heavy variable 1–2	27.03	0	− 7.02	4.01e−02
*IGKC*	Immunoglobulin kappa constant	735.33	6.67	− 6.76	9.58e−03
*IL19*	Interleukin 19	4.88	67.71	3.85	5.34e−03
*KRT17*	Keratin 17	202.77	786.77	1.96	4.01e−02
*KRT78*	Keratin 78	156.51	656.65	2.07	1.58e−02
*LTF*	Lactotransferrin	176.91	19.45	− 3.29	2.56e−03
*MAL*	mal, T cell differentiation protein	16.72	220.79	3.74	5.34e−03
*PARP15*	Poly(ADP-ribose) polymerase family member 15	112.10	6.77	− 3.95	2.65e−02
*PLA2G2A*	Phospholipase A2 group IIA	20,672.08	6669.91	− 1.63	1.08e−03
*SPDEF*	SAM pointed domain containing ETS transcription factor	76.81	316.64	2.05	1.56e−02
*SPRR1B*	Small proline rich protein 1B	123.08	485.14	1.98	3.91e−02
*SPRR3*	Small proline rich protein 3	310.77	2203.18	2.83	9.58e−03
*TFF3*	Trefoil factor 3	110.21	480.32	2.12	1.70e−02
*TLE1*	TLE family member 1, transcriptional corepressor	93.26	235.54	1.33	2.51e−02
*TMPRSS11E*	Transmembrane serine protease 11E	17.59	173.33	3.33	2.30e−02
*ZBTB16*	Zinc finger and BTB domain containing 16	39.26	278.59	2.83	6.78e−03

**Table 4:** List of enriched biological processes in PANTHER.

Biological process	Genes enriched	FDR
**Upregulated genes**		
Keratinization	*FLG, SPRR1B, KRT17, SPRR3, KRT78*	1.83 × 10^−3^
**Downregulated genes**		
Regulation of B cell activation	*IGHV1-2, IGKC, ATM, IGHG1, BANK1*	1.49 × 10^−3^
Negative regulation of immune system process	*IGHV1-2, IGKC, ATM, IGHG1, BANK1, LTF*	2.21 × 10^−3^
Regulation of inflammatory response	*PLA2G2A, IGHV1-2, IGKC, ATM, IGHG1, DNASE1L3*	1.64 × 10^−3^

To each gene in Gene Symbol column was associated the corresponding name in Gene Name column with the bitr function of the cluster Profiler package of Bioconductor. The expression of each gene in healthy individuals and COVID-19 patients was highlighted in the columns Healthy Expression and COVID-19 Expression, respectively. The column Fold Change shows the changes in the expression level between healthy and COVID-19 individuals while the q-Value column shows significance of the difference in the dataset (q < 0.05).

For each of the most specific biological processes found with PANTHER, the differentially expressed genes that are included were highlighted: a pathway is enriched for the upregulated genes and 5 genes are included; 3 pathways are enriched for the downregulated genes and 6 genes are included. The False Discovery Rate (FDR) of each pathway is lower than 0.05.

**Figure 2:**
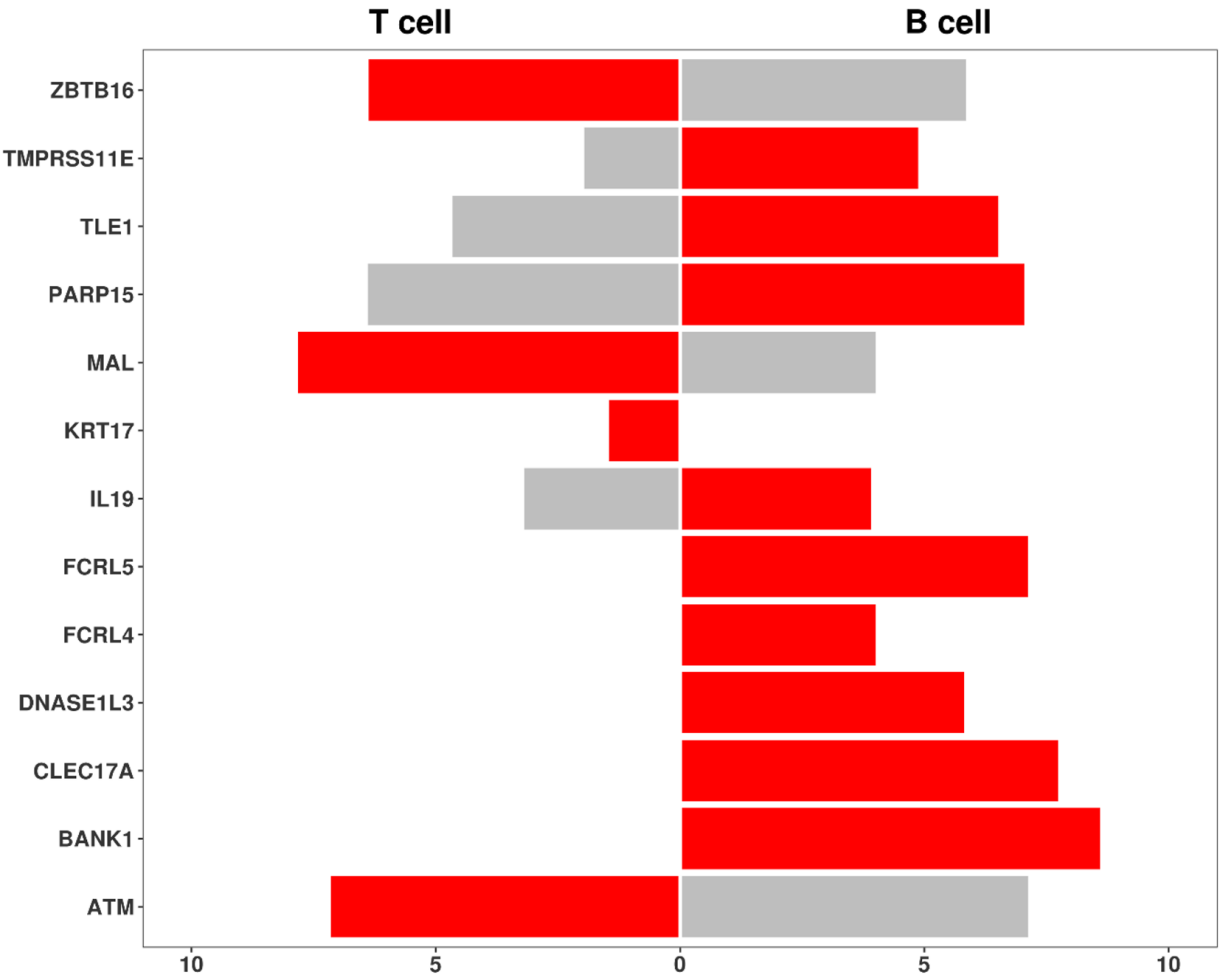
Gene expression in T and B cells retrieved in Human Protein Atlas database. The bars represent the mRNA expression level of each gene in T cells (on the left) or in B cells (on the right). The bar is red in the cells in which the gene is more expressed, grey otherwise.

We then retrieved from the Human Protein Atlas database^17^ (Human Protein Atlas available from^18^) the mRNAs expression level of the genes found in our analysis in B and T cells. As depicted in red bars of the pyramid plot in Fig. 2, the most of the genes (*TMPRSS11E, TLE1, PARP15, IL19, FCRL5, FCRL4, DNASE1L3, CLEC17A* and *BANK1*) are more expressed in B cells than in T cells. Conversely, only *ZBTB16, MAL, KRT17* and *ATM* are more expressed in T cells. Moreover, no expression was detected for *FCRL5, FCRL4, DNASE1L3 CLEC17A* and *BANK1* in T cells while *KRT17* is no expressed in B cells.

## Discussion

The eye has been considered as a potential site for SARS-COV-2 viral infection and dissemination^10,19^. Moreover, involvement of the eye seems to be related more likely to severe forms of COVID-19 disease and often precedes the systemic symptoms or even is the only sign of the disease^15^. In our sample all diseased subjects had a severe SARS-CoV-2 infection; most of them had pneumonia (78.9%) and 70% of cases died with a mean of 26 ± 12 days of hospitalization.

Our study aimed to provide a panel of gene expression in tears of patients affected by SARS-CoV-2 infection comparing with healthy subjects to understand the gene expression pattern in the eye, which can be a starting point site of infection, as well as a concomitant involved organ of the disease. Given the protective function of tear film preserving the homeostasis and health of the conjunctiva and the avascular cornea, abnormalities in concentrations of proteins and inflammatory mediators in lacrimal secretions have been observed in infections, surgery and trauma. Indeed, antimicrobial factors have been well described in tears including lysozyme, lactoferrin, transferrin, ceruloplasmin, IgA, IgG, IgE, complement, glycoprotein, and anti-proteinase, which are found in the aqueous layer of the tear film. As already reported, immunoglobulins play a key role in defense of bacterial, viral, and parasitic infection. IgA, which is the primary immunoglobulin found in tears produced by conjunctiva and lacrimal gland, usually increases during infectious or inflammatory conditions of conjunctiva^20^. Moreover conjunctivitis has shown a rise in inflammatory mediators (IL-1β, TNF-α, and MMP-9) and the activation of proinflammatory mitogen-activated protein kinase (MAP-K) pathways^21^. In detail, tear film has three major layers, such as the inner mucin layer, the middle aqueous layer and the outer lipid layer^22,23^. The inner layer, formed by mucins secreted mainly by the goblet cells in the conjunctival epithelium, has stabilizing role of aqueous layer. It is composed by immunoglobulins, urea, salts, glucose, and proteins as well. The aqueous layer, essential for maintaining hydration and health of the ocular surface, contains proteins, metabolites, inorganic salts, glucose, oxygen, and electrolytes (magnesium, bicarbonate, calcium, urea). The lipid layer, fundamental for controlling tear evaporation, contains cholesterol, wax esters, fatty acids, and phospholipids^23,24^. Other tear film components include lysozyme with its bacteriolytic role, lactoferrin that is able to sequester iron from the bacteria thus stopping their growth. Mucins and glycoproteins secreted by goblet cells have a known role in ocular defense from external environment, preventing attachment of pathogens to ocular surface. In our sample, RNA-seq analysis revealed 25 genes differing statistically between healthy individuals and patients with a diagnosis of COVID-19. In detail, in the COVID-19 subjects 13 genes were upregulated, while 12 genes were downregulated.

We found that different genes involved in the function of B cells were downregulated, as also confirmed by Panther analysis that evidenced that the biological process “Regulation of B cell activation”, but also “Regulation of inflammatory response” and “Negative regulation of immune system process”, were enriched for the downregulated genes. Interestingly, a previous report indicated that lymphocyte B decreased in COVID-19 patients^25^. Among these down-regulated genes in COVID-19 group, we found *IGKC*, *IGHG1* and *IGHV1-2*, encoding for immunoglobulin constant and variable chains related to immune response. Previous findings, have reported the *IGKC* downregulation in the tears of patients affected by other diseases, including bilateral keratoconus^26^. *IGHG1* decrease was reported also in Fuchs endothelial corneal dystrophy^27^. Moreover, also the genes *FCRL4* and *FCRL5*, that are receptors for IgA and IgG, respectively, were downregulated and have also a role in viral infections^28,29^.

*DNASE1L3*, that was downregulated in COVID-19 group, encoded for DNase γ, member of DNase I family of endonucleases. It was found to be expressed in germinal center B cells and stimulated B cells. It is involved in the somatic hypermutation of immunoglobulin variable region genes that occurs in the germinal center B cells during immune responses, and then contributed to the immunoglobulin V gene diversification^30,31^. *DNASE1L3* is also involved in the release of cytokines after activation of the inflammasome^32^.

*BANK1* also was found to be downregulated in the tears of COVID-19 patients. It is a positive regulator of B cell signaling through the induction of calcium mobilization after the activation of B-cell antigen receptor^33^. Also *CLEC17A*, expressed in B cells with function of adhesion to epithelial cells^34^, was downregulated in our analysis.

After sequencing we reported a downregulation of the ATM gene which synthesizes serine/threonine-protein kinase that act in the cell following DNA damage^35^. ATM has an important role in both T and B cells function. In particular, the loss of ATM in B cells caused the reduction in germinal center frequency and size in response to immunization and apoptosis of B cells^36^. Moreover, ATM is involved also in T cell development^37^. Moreover, it was found that RNA viruses can cause DNA damage and genetic instability in host cells modulating components of the DNA damage response, such as ATM^38^. ATM gene mutation were found in ocular adnexal marginal zone B-cell lymphomas and uveal melanoma^39–41^. In addition ATM gene inhibition reduces herpes virus corneal infection and particularly epithelial infection and stromal disease.

We found the upregulation of *TLE1*, *MAL* and *ZBTB16* in the tears of COVID-19 patients. The TLEs family is composed by co-repressors expressed in T cells where they are required for CD8^+^ T cell lineage choice^42^. MAL is involved in apical transport of proteins in polarized epithelial cells^43^. MAL has been shown to be implicated in lytic plaque formation and viral spread in oligodendrocytes infected with Herpes simplex virus type 1^44^. It is involved also in T cell functions^45,46^. *ZBTB16* is a gene also known as PLZF (promyelocyticleukemia zinc finger). It is important in the function and development of immune system and may enhance T cell responses^47^.

Our seq analysis revealed a significant upregulation of *IL19*. The upregulation of IL-19 was already found in blood of COVID-19 patients^48^. Li et al. described the gene expression profile from patients with Th cell-mediated autoimmune noninfectious uveitis and some cytokines, including IL-19, were found to be highly expressed proving the inflammatory status of the eye^49^. The upregulation of such inflammatory interleukin in patients with the coronaviridae disease shows an inflammation of conjunctiva. Interestingly, IL-19 is produced by macrophages, B-cells but also by keratinocytes, and interestingly, it can also act on keratinocytes, suggesting to be involved also in keratinocyte hyperproliferation^50,51^. This aspect is particular interestingly, considering that we found the upregulation of genes involved in keratinization as confirmed also by the enrichment of the same biological process.

We also found an upregulation of keratin 17 and keratin 78 expression levels in tears of our enrolled patients. A similar *KRT17* upregulation was detected by Kulkarni et al.^52^ in limbal epithelial stem cells of diabetic patients using deep sequencing analysis if compared with healthy individuals. The authors speculated that KRT17 dysregulation could be involved in the typical corneal modifications of diabetic subjects. From our analysis *KRT17* and *KRT78* were upregulated likely due to their potential role in ocular surface modifications during SARS-CoV-2 infection.

Our findings showed a significant increase in *FLG* levels compared to the healthy subjects. FLG has also an important role in maintaining the integrity of stratum corneum of epidermidis and its loss-of-function mutations have been associated with atopic dermatitis, due to a reduced skin hydration and skin barrier function^52^. A previous study indicated that *FLG* was not expressed in normal conjunctiva, while it increased in moderate and severe forms of parakeratinization of the conjunctiva^53^.

We found the upregulation of both *SPRR1B* and *SPRR3.* They are part of the small proline-rich proteins (SPRRs), which constitute cornified cell envelope precursors^50,54^. SPRR1B is a stress-induced transcript on the ocular surface that was shown to be upregulated in conditions of pathologic keratinizationin, in both evaporative and immune-mediated, aqueous-deficient dry eye disease. SPPR1B can be also considered a biomarker for pathology such as Sjögren's syndrome and for the study of the molecular mechanisms of squamous metaplasia. Squamous metaplasia causes pathologic keratinization of the ocular surface in response to disease processes that are autoimmune mediated or caused by infection. Moreover, pro-inflammatory cytokines induced the expression of SPRR1B^55^.

*SPDEF*, upregulated in our analysis, is a transcription factor that promotes the differentiation of goblet cells in the conjunctiva epithelium^56^. Goblet cells produce and secrete mucins, needed to lubricate the ocular surface. Goblet cell secretions are essential in order to maintain tear stability and ocular surface homeostasis. Goblet cells in the conjunctiva play an essential immunomodulatory role^57^. Increased expression of SPDEF was associated to goblet cell hyperplasia^58^ and upregulation of goblet cell-associated genes^59^. Atopic keratoconjunctivitis was associated with goblet cell hyperplasia^60^.

*TFF3* was found to be upregulated in our analysis. It is expressed in various ocular tissues, also in goblet cells and has a role in corneal wound healing^61^. Its increase in tear film was found after inflammatory factors or ocular surface damage, such as those in dry eye disease, in experimental models^62^. It increased also in herpetic keratitis^63^.

*LTF*, produced in the acinar cells of the lacrimal gland, is normally present in tears of humans and is not dependent on age and sex. It has been proved that LTF is reduced in some ocular and systemic disease such as dry eye-related keratopathies, herpes simplex keratitis, chronic irritative conjunctivitis keratoconjunctivitis sicca^64^.

*PLA2G2A*, which is downregulated in tears of our diseased sample of patients, has been previously described to be involved in tear film stability and the consequent integrity of tear ocular surface^65^.

*ALOX15B* is a lipoxygenase enzyme. It was shown that LPS bacteria and the proinflammatory cytokines may induce overexpression of ALOX15B^66^. It represents the predominant 15-LOX protein in human cornea, and its product induced apoptosis in a dose dependent manner^67^.

Type II transmembrane serine proteases (TTSPs) have the ability to cleave surface proteins of viruses, including SARS-CoV-2 and influenza viruses, leading to the viral invasion. In particular, SARS-CoV-2 can bind TMPRSS2 of the host cell. This protease facilitates the viral attachment to the surface of targets cells by cleavage and priming of a spike protein of coronaviruses (S protein), at the S1/S2 and the S2’ site. This process seems to be the first essential step for the virus entry in the human body, thus demonstrating a key role of TMPRSS2 activity on SARS-CoV-2 spread^68^. Interestingly, in our RNA-seq analysis we found *TMPRSS11E* upregulated in tears of COVID-19 patients, leading to the hypothesis of a possible additional role of this protease in the Sars-Cov-2 entry in the host cells. Indeed, it was demonstrated that TMPRSS11E also known as DESC1, activated the S proteins of emerging Coronavirus^69^.

*PARP15* is a neurodegeneration mediator and its inhibition seems to improve corneal epithelial innervation and wound healing in diabetic rats^70^. It is known that PARPs may play a role in inflammation and virus infections, indeed coronavirus may have the ability to reverse ADP ribosylation induced by PARP in order to counteract host-virus defense response^71,72^.

We can speculate that the down or up regulation of some of the investigated genes already known as involved in ocular inflammation processes are related to the presence of the virus on the ocular surface. In particular the up regulation of IL19 gene, associated to non infectious uveitis and conjunctivitis could be related to SARS-CoV-2 infection in our patients. TFF3 also found to be upregulated in our sample has already been related to corneal inflammation in dry eye disease herpetic keratitis. The inhibition of ATM gene, downregulated in our study, has been found related to a reduction of herpes virus corneal infection.

The main limitation of our study is the lack of a sub analysis differentiating COVID-19 patients with and without conjunctivitis to correlate ocular clinical conditions and the RNA seq analysis.

**Figure 3:**
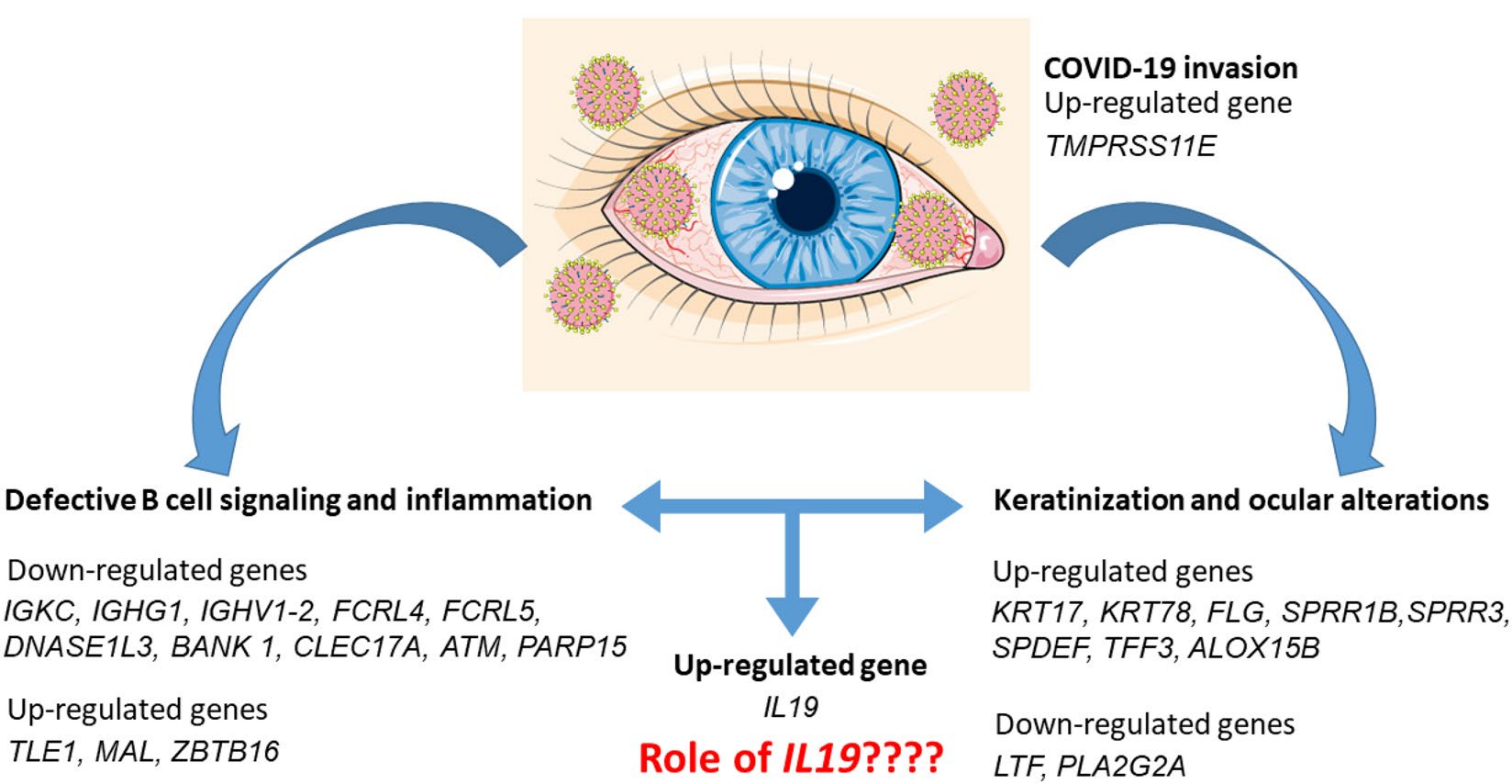
Schema of the down- and up-regulated genes and the processes in which they are involved. The trancriptomic analysis suggested a defective function of B cells and a process of keratinization. IL-19 may link the process, being involved also in keratinocyte proliferation. The figure was made taking the images from Servier Medical Art ( available at http://smart.servier.com/), licensed under a Creative Commons Attribution 3.0 Unported License ().

In conclusion, our study reported in tears of patients suffering from SARS-CoV-2 infection a downregulation of genes involved in B cell signaling, while the genes involved in keratinization were upregulated (Fig. 3). These results are particularly interesting because to our knowledge this is the first study that suggest a possible molecular mechanism responsible of the keratoconjunctivitis reported in patients affected by COVID-19^73,74^. It would be interesting to investigate if IL19 increase may link the inflammation process with the keratinization.

## Materials and methods

### Patients enrollment

In this observational study, approved by our Institutional Review Board (prot. n. 1497/22.05.2020), a total of 19 patients with a diagnosis of COVID-19 (COVID group) were enrolled at the Infectious diseases department and the Internal Medicine Clinic, at the “SS. Annunziata Hospital” of Chieti-University G. D’Annunzio, Chieti-Pescara, Italy, from April 2020 to May 2020. A group of healthy patients (n = 20; Healthy group) with negative nasopharyngeal/throat swabs were enrolled and were considered as controls. The study adhered to the tenets of the Declaration of Helsinki and a written informed consent was obtained from all participants.

### Study population

The inclusion criteria (COVID group):

- A confirmed diagnosis of COVID-19 (diagnosis confirmed by positive detection of SARS-CoV-2 RNA from nasopharyngeal/throat swabs by real time (RT)-PCR);
- Age ≥ of 18 years.

The inclusion criteria (Healthy group):

- Age ≥ of 18 years;
- No ocular and systemic diseases;
- No assumption of drugs.

The exclusion criteria (COVID group and Healthy group):

- Pregnant women;
- Any form Ocular surface diseases preceding Covid-19 diagnosis, Glaucoma, history of anterior segment inflammation, previous penetrating ocular trauma;
- Ocular surgeries within previous 6 months;
- Topical therapies;
- History of ocular allergy.

The following anamnestic data about all patients enrolled were collected:

- Demographic data (age, sex, race);
- Duration of the hospitalization;
- Pre-existing illness (cardiac, neurologic, nephrologic and auto-immune diseases, hypertension, diabetes, allergies, obesity);
- Smoking history;
- Relatives affected with Covid-19;
- Department of recovery (Intensive Care Unit or other department);
- Medical treatments (on admission and during the hospitalization);
- Laboratory findings;
- Symptoms (shortness of breath, cough, fever, myalgia, pneumonia);
- SaO2;
- Evidence of conjunctivitis (presence of bilateral conjunctivitis was defined as red eyes: macroscopic signs of conjunctival congestion and was confirmed by using a portable slit lamp (SL‐17; Kowa Ltd., Tokyo, Japan) and if present the onset of the ocular disease was registered: pre-admission, on admission, during hospitalization);
- Necessity of non-invasive (facemask oxygen)/invasive mechanical ventilation;
- Clinical outcomes (recovery, discharge from hospital, death).

### Tear sampling

Tear secretion was measured by using the Schirmer’s Test (Schirmer strips; Whatman, Maidstone, UK). Schirmer’s papers have been kept in the lower lid margin for 5 min. Tear secretion was measured as the length of the wet strip (in millimeters). Schirmer’s tear fluid collection was carried out simultaneously in both eyes. The paper strips from eyes were placed in Eppendorf tubes containing 500 μl of water with RNase inhibitors (Diethyl pyrocarbonate (DEPC)-treated water, Ambion, CA, United States) and strongly vortexed for 5 min and frozen in dry ice. Upon arrival to the laboratory both samples have been stored at − 80 °C.

### Real time-PCR

SARS-COV-2 mRNA expressions in tear samples were assessed by RT-PCR. For this purpose, total RNA was isolated utilizing the Total RNA Purification kit (NorgenBiotek Corp., Thorold, ON, Canada). The M-MLV Reverse Transcriptase reagents (Applied Biosystems, Foster City, CA, USA) were utilized to produce cDNA. Real-time PCR was performed with the Mastercycler ep realplexrealtime PCR system (Eppendorf, Hamburg, Germany). Commercially available TaqMan Gene Expression Assays (Applied Biosystems) and the TaqMan Universal PCR Master Mix (Applied Biosystems) were utilized in agreement with the standard protocols^75^. Beta-2 microglobulin (B2M Hs99999907_m1; Applied Biosystems, Foster City, CA, USA) was utilized for template normalization. RT-PCR was analyzed in three independent experiments; duplicate determinations were obtained for each sample.

### RNA extraction and library preparation

The library was prepared in according with Illumina protocols and how already described^76^. Briefly, the total RNA was extracted with the Maxwell RSC simplyRNA Cells Kit (Promega, Madison, WI, USA) following the manufacturer’s instructions. The library preparation was carried out according to the TruSeq RNA Exome protocol (Illumina, San Diego, CA, USA)^77^.

The cDNA was synthesized with the SuperScript II Reverse transcriptase (Invitrogen, Carlsbad, CA, USA) and the 3’end was later adenylated. The library was amplified with PCR and validated. Two steps of hybridization followed the first PCR and later one more PCR was performed. The MiSeq Instrument (Illumina) was used to sequence the libraries.

### Bioinformatics analysis

The obtained raw data was checked for quality using the fastQC tool and the needed trimming for adapters and low quality bases scores was performed by Trimmomatic (Usadel Lab, Aachen, Germany)^78^ (version 0.38). The reads were than aligned to the version GRCh38 of the human reference genome with the Spliced Transcripts Alignment to a Reference (STAR) RNA-seq aligner^79^. After sorting the reads with STAR, the counting of the reads was made using the htseq-count python package^80^. The differentially expressed genes were finally obtained using the Bioconductor package DESeq2 in R language^81^. The Benjamini–Hochberg post-hoc procedure with threshold 0.05 was used to filter out the false positives. The bitr function of the clusterProfiler package of Bioconductor associated each gene symbol with its corresponding name^82^. Panther was then used to enrich the genes with the biological processes in which they are involved. Finally, the Human Protein Atlas database was used to find the mean level of expression of the mRNAs coding for these genes in B cells and in T cells. After logaritmic conversion, the values were depicted in R using the function ggplot2^83^.

## Data Availability

The datasets generated during and/or analyzed during the current study will be available from the corresponding author on reasonable request.

## Abbreviations

COVID-19: Coronavirus disease 2019
NGS: Next generation sequencing
PCR: Polymerase chain reaction
